# Lumen imaging in calcified coronary arteries using high-resolution Gd-enhanced color K-edge imaging with spectral photon-counting CT: a phantom study

**DOI:** 10.1186/s41747-026-00720-3

**Published:** 2026-05-06

**Authors:** Magdalena M. Dobrolinska, Niels van der Werf, Marcel Greuter, Antoine Robert, Marjorie Villien, Hugo Lacombe, Dominika Suchá, Angele Houmeau, Philippe Coulon, Sara Boccalini, Philippe Douek, Salim Si-Mohamed

**Affiliations:** 1 Department of Radiology, University Medical Center Groningen, University of Groningen, Groningen, The Netherlands; 2 Division of Cardiology and Structural Heart Diseases, Medical University of Silesia in Katowice, Katowice, Poland; 3 Philips Healthcare, Best, The Netherlands; 4 University of Lyon, INSA-Lyon, Université Claude Bernard Lyon 1, UJM-Saint Etienne, CNRS, Inserm, CREATIS UMR 5220, U1206, Villeurbanne, France; 5 Philips Healthcare, Suresnes, France; 6 Department of Radiology and Nuclear Medicine, University Medical Center Utrecht, Utrecht, The Netherlands; 7 Radiology Department, Department of Cardiovascular and Thoracic Radiology, CHU Cardiologique Louis Pradel, Louis Pradel Hospital, Bron, France

**Keywords:** Calcium, Computed tomography angiography, Coronary arteries, Gadolinium, Phantoms (imaging)

## Abstract

**Objective:**

Conventional coronary computed tomography angiography (CCTA) lumen assessment is hampered by the similar attenuation of iodine and calcium. We assessed the lumen for Gd-enhanced CCTA on high-resolution color Gd K-edge imaging using a clinical spectral photon-counting computed tomography (SPCCT) prototype in an anthropomorphic phantom.

**Materials and methods:**

A hollow cylindrical coronary artery phantom (10-mm outer diameter, 5-mm inner diameter) containing five cylindrical calcifications of different densities (75, 100, 200, 400, and 800 mg/cm^3^ hydroxyapatite, deemed very low, low, medium, high, and very high, respectively) and equal size was scanned on a clinical SPCCT prototype using a clinical CCTA protocol. The artery model was filled with Gd mixed with saline to achieve 400 HU at 70 keV. The luminal area was compared with the physical area (*i.e*., 19.6 mm^2^), and spectral results were compared to conventional acquisition.

**Results:**

In the absence of calcification, physical lumen size was overestimated by 7% and 16% on color Gd K-edge and conventional images, respectively. In the presence of calcification, only color Gd K-edge images enabled the measurement of the lumen, *i.e*., 22.25 ± 0.43 mm^2^, 22.85 ± 1.77 mm^2^, 14.63 ± 1.17 mm^2^, and 15.37 ± 0.78 for very low to very high calcium density, respectively. Largest underestimation (-26%) and overestimation (16%) were shown for the high- and low-density calcification in comparison to physical size, respectively.

**Conclusion:**

Color Gd K-edge imaging with Gd-enhanced CCTA outperformed conventional imaging in assessing the coronary lumen in both noncalcified and calcified vessels, whilst accuracy depends on the presence and density of calcifications.

**Relevance statement:**

In a phantom study, high-resolution color Gd K-edge imaging targeting Gd enabled accurate visualization of the coronary lumen in both calcified and noncalcified arteries, outperforming conventional CT angiography. In the presence of calcifications, only color Gd K-edge imaging provided objective lumen assessment, with accuracy influenced by calcium extent and density.

**Key Points:**

- Color Gd K-edge Gd-enhanced CCTA using SPCCT is feasible in a coronary artery phantom.
- Color Gd K-edge imaging improves lumen assessment in calcified vessels.
- Color Gd K-edge imaging potentially enhances diagnostic precision and treatment planning for patients with coronary artery disease.

**Graphical Abstract:**

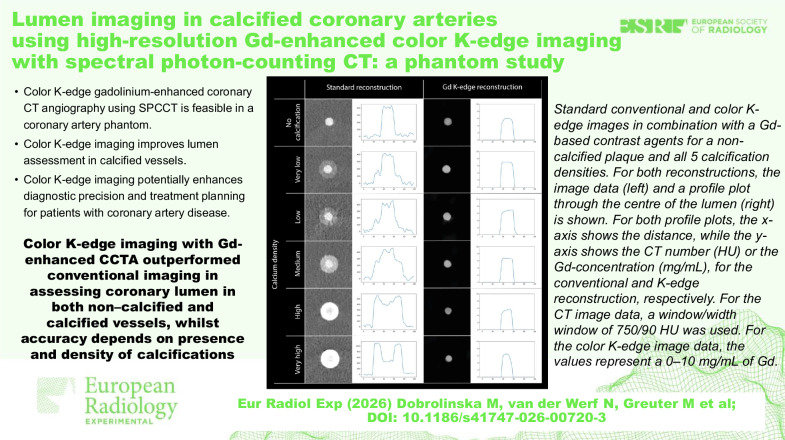

## Background

Noninvasive coronary computed tomography angiography (CCTA) has emerged as a standard diagnostic method for coronary artery disease [1, 2]. However, challenges such as increased noise levels hampering the contrast between coronary artery plaque and lumen, as well as blooming artifacts resulting from high-density calcifications, remain significant limitations of CCTA performed on conventional computed tomography (CT) systems.

Introduction of the spectral photon-counting computed tomography (SPCCT) system presents a pioneering solution to address current shortcomings in noninvasive cardiovascular diagnosis [3–6]. This advanced technology utilizes smaller detector elements that directly convert individual photons into electronic signals. In contrast to conventional CT systems that integrate values from multiple photons, SPCCT enables the quantification of single photons on much smaller detector elements, thereby significantly enhancing spatial resolution, and minimizing blooming artifacts. The distinguishing feature of SPCCT lies in its capacity to not only count photons directly and individually but also classify them into energy bins. Importantly, only photons exceeding the background electronic noise are counted, and as a result, it eliminates the impact of electronic noise. For systems with more than two energy bins, so-called K-edge imaging is possible, whereby materials can be identified based on specific K-edge energy [7]. Previously, this type of imaging was referred to as ‘multicolor’ or ‘color’ CT imaging [8–14].

Color K-edge imaging uses the fact that a bounding energy of K-shell electrons from the atomic K-shell is discriminative for each element. Therefore, knowing that only photons with this specific K-edge shell energy are counted, an element with this specific energy can easily be distinguished from others. It is noteworthy to mention that a K-edge energy of a contrast agent should lie within the clinical CT energy range (*i.e.,* ~40–140 keV), which is not the case for iodine (*i.e*., 33.2 keV) [14]. The limited separability of iodine and calcium in conventional CT arises from insufficient spectral contrast in patient-size imaging. Iodine’s K-edge lies below the clinically usable energy range, and low-energy photons are strongly attenuated, reducing material-specific spectral information. This limitation subsequently manifests as overlapping HU values between iodine and calcium. This stands in contrast to Gd, with a K-edge energy of 50.2 keV. Despite the fact that Gd has been previously used for various color K-edge imaging applications [15–19], as well as being used recently in humans [20], the knowledge of its feasibility in the context of coronary artery lumen is scarce.

Therefore, the aim of this study is to assess the potential of coronary artery lumen evaluation using color Gd K-edge imaging in combination with Gd for both noncalcified and calcified vessels from CCTA using an SPCCT system.

## Methods

### Phantom

**Fig. 1:**
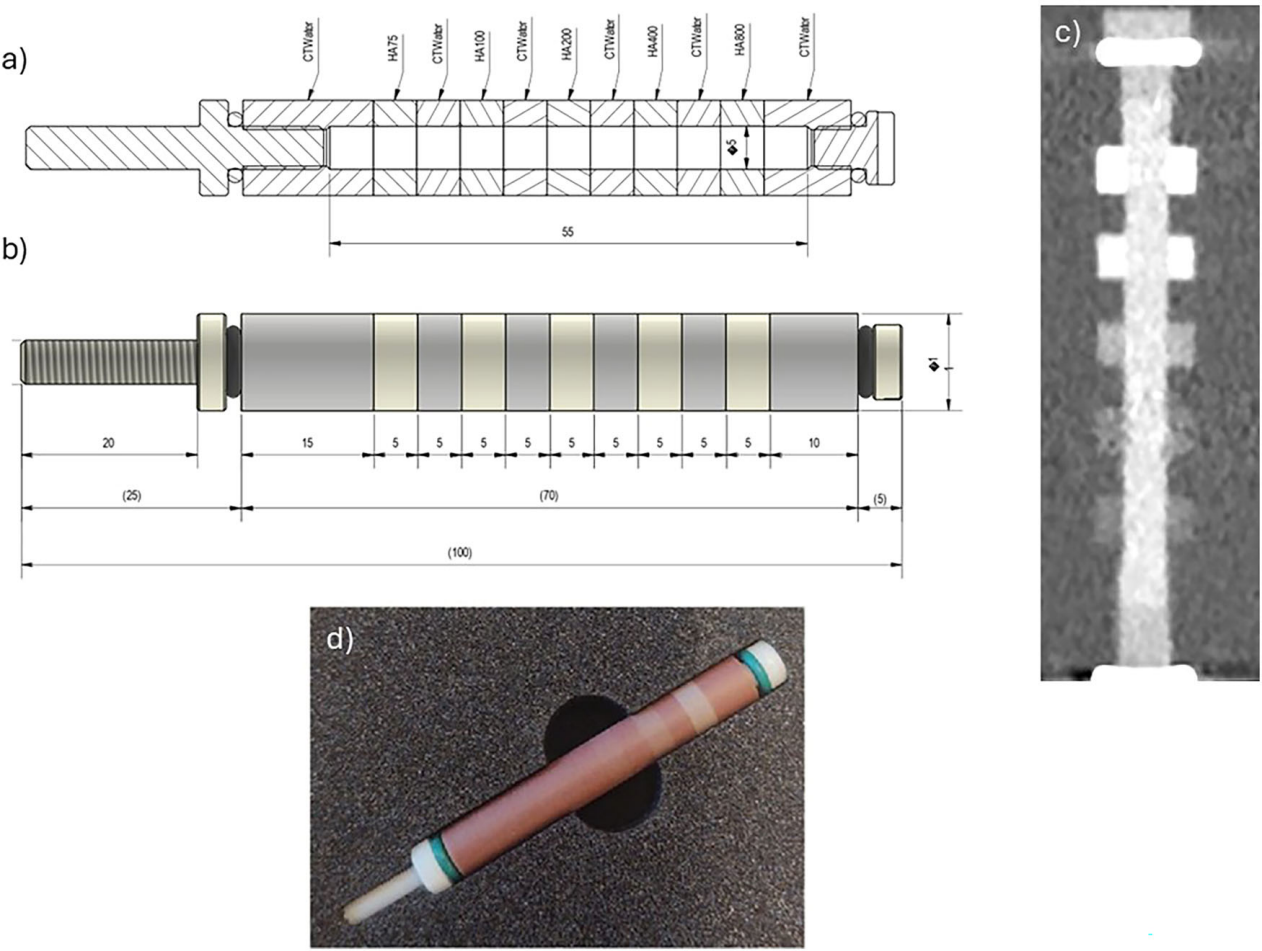
Overview of hollowed coronary artery phantom with five calcifications of difference densities designated as: very low (75 mg/cm^3^ HA), low (100 mg/cm^3^ HA), medium (200 mg/cm^3^ HA), high (400 mg/cm^3^ HA), and very high (800 mg/cm^3^ HA) calcium density (**a**, **b**). The inner diameter of the phantom is 5 mm. In addition, the longitudinal photon-counting CT image of the coronary artery phantom (**c**), as well as the corresponding phantom image (**d**), are presented for visualization. HA, Hydroaxyapatite

A coronary artery model consisting of five hollow cylindrical calcifications of different densities (75, 100, 200, 400, and 800 mg/cm^3^ hydroxyapatite, deemed very low, low, medium, high, and very high, respectively), and of equal size (outer diameter 11 mm, inner diameter 5 mm, and length 5 mm) was scanned. The lumen of the artery model (diameter 5 mm, in-plane area of 19.63 mm^2^) allowed for the insertion of contrast media (Fig. 1). The coronary artery model was placed at the center of an anthropomorphic thorax phantom (QRM thorax, QRM) inside a water compartment (diameter of 100 mm). To imitate a large patient size, a fat extension ring was added to the phantom (Extension Ring L, QRM), increasing the outer dimensions to 400 mm × 300 mm [20]. The coronary artery model was filled with a mixture of saline and Gd (Gadoteridol, ProHance, Bracco Imaging) at a concentration reaching 400 Hounsfield Units (HU) at 70 keV, which reflects the clinical range for CCTA when iodine is used as a contrast agent [21, 22]. The scan was repeated five times with a small translation and rotation of the phantom to account for inter-scan variability.

### Data acquisition and reconstruction

Data acquisition was performed on a clinical spectral photon counting CT (SPCCT) prototype (Philips Healthcare), with detectors of 2-mm-thick cadmium zinc telluride, bonded to Philips’ proprietary ChromAIX2 application-specific integrated circuit [23]. Energy thresholds have been set for color Gd K-edge imaging with Philips proprietary spectral decomposition algorithm. The 5 energy thresholds define 5 energy bins. Energy-discriminating thresholds were set to 30, 51, 62, 72, and 81 keV for Gd K-edge (*i.e.*, 50.2 keV) detection. The counts in each of these energy bins are used to calculate the contributions of the photoelectric effect, Compton scatter, and color Gd K-edge to the total X-ray attenuation. To create the conventional CT image from the same acquisition, all counts, *i.e.*, above the 30 keV threshold, are used. Technical details concerning the prototype systems are explained elsewhere [24]. For data acquisition, a routine clinical CCTA protocol was used (Table 1).

**Table 1:** Summary of data acquisition and reconstruction parameters

Technique	Axial
Tube voltage [kVp]	120
Tube current time product [mAs]	330
Automatic exposure control	Off
Energy bin threshold	30–51–62–72–81 keV
Focal spot	0.6 × 0.7 × 2 (dual focal spots)
Collimation [mm]	64 × 0.275
Field of view [mm]	200
Rotation time [s]	0.33
Slice thickness [mm]	0.5
Reconstruction algorithm, level	iDose, 6
Matrix size [pixels]	1024
Reconstruction filter	HRB
Repetitions	5

### HU and color Gd concentration measurement

For each of the five repetitions, CT numbers (HU), Gd concentration (mg/mL), and their standard deviations (noise) were derived from conventional and color Gd K-edge of Gd images, respectively, in the complete lumen at the calcified and noncalcified slice. To exclude any edge effects from the transition of lumen to background material, a threshold of 80 HU or 1 mg/mL was used for the conventional and color Gd K-edge image data, respectively. The analysis was performed with an in-house developed Python script (Python version 3.8,https://gitlab.com/nrvdwerf/ct_phantomanalysis/) [25].

### Lumen area calculations

As a second step, lumen analysis was performed at a noncalcified slice (at the center of the artery, in between the low and medium calcification) and at the level of the five calcifications. For both, cross sectional coronary lumen area was derived. Coronary artery lumen was analyzed with an in-house developed Python script (Python version 3.8) [25]. In addition, the percentage of under- or overestimation of lumen area as compared to the reference was calculated, with the reference lumen area defined as the reference physical cross-sectional lumen area of 19.63 mm^2^.

### Noise, signal-to-noise, and contrast-to-noise ratio

Three quality metrics were calculated. First, noise values on conventional (HU) and color Gd K-edge (mg/mL) images were measured. Second, signal-to-noise ratio (SNR) and contrast-to-noise ratios (CNR) for the coronary artery lumen filled with Gd at the noncalcified slice/location were calculated as follows:

$${{SNR}}_{{HU}}=\frac{{Lumen}{\left({HU}\right)}_{{mean}}}{{{Background}\left({HU}\right)}_{{SD}}}$$

$${{SNR}}_{{mg}/{mL}}=\frac{{{Lumen}\left({mg}/{mL}\right)}_{{mean}}}{{{Background}\left({mg}/{mL}\right)}_{{SD}}}$$

$${{CNR}}_{{HU}}={LumenGadolinium}{\left({HU}\right)}_{{mean}}-{Background}$$

$$	{{CNR}}_{{mg}/{mL}}= \\ 	\frac{{{LumenGadolinium}\left({mg}/{mL}\right)}_{{mean}}-{{Background}\left({mg}/{mL}\right)}_{{mean}}}{{{Background}\left({HU}\right)}_{{SD}}}$$

where the Background (HU)_mean_ and Background (HU)_SD_ were defined as the mean CT number and SD of the CT numbers for conventional acquisitions, respectively, within a region-of-interest of 450 voxels (15 × 30) in the water compartment. Accordingly, for color Gd K-edge images, Background (mg/dL)_mean_, and Background (mg/dL)_SD_ were defined as the mean and SD of the concentrations, respectively, within a region-of-interest of 450 voxels (15 × 30) in the water compartment.

### Statistical analysis

Continuous variables were reported as mean and SD [26]. The mean lumen area was compared to the reference defined as a coronary artery phantom lumen area. The lumen area, noise levels, SNR, and CNR were compared using Student’s *t*-test. Analysis of variance (ANOVA) was used for repeated measurements of lumen area and Gd concentrations. All statistical analyses were conducted using SPSS 28 (IBM). Statistical significance was defined as *p* < 0.05.

## Results

### Gd concentration

**Fig. 2:**
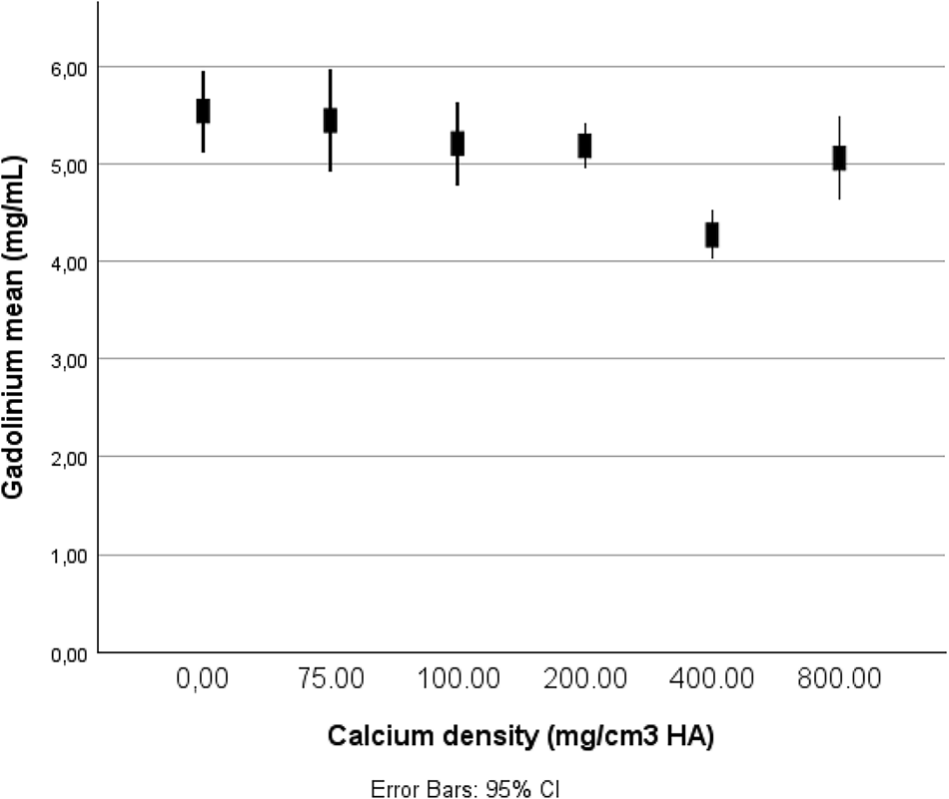
Summary of Gd concentration at the level of noncalcified artery, very low, low, medium, high, and very high-density calcifications, respectively. All measurements were acquired from color Gd K-edge images

In the absence of calcification, the mean Gd concentration was 5.54 ± 0.33 mg/mL. In the presence of very low, low, medium, high, and very high-density calcifications, the Gd concentration was 5.44 ± 0.43, 5.21 ± 0.35, 5.18 ± 0.17, 4.27 ± 0.20, 5.06 ± 0.34 mg/mL, respectively. Gd concentrations were significantly lower in the presence of high and very high-density calcifications (*p* < 0.001) (Fig. 2).

### Image quality metrics

Values of noise, SNR, and CNR are summarized in Table 2. Both color Gd K-edge derived SNR and CNR in the absence of calcifications showed significant increases of 57% and 59%, in comparison to the conventional reconstruction, respectively (*p* < 0.001).

**Table 2:** HU values, Gd concentration, SNR, and CNR for conventional and color Gd K-edge imaging at the level of noncalcified arteries

Conventional images				
HU	Background mean	Background SD	SNR-HU	CNR-HU
348.8 ± 2.8 (346.6–356.9)	13.6 ± 3.4 (9.9–16.6)	24.3 ± 1.6 (21.9–25.8)	14.43 ± 1.12 (13.47–16.3)	13.9 ± 1.1 (12.8–15.6)

Color Gd K-edge images				
Gd [mg/dL]	Background mean	Background SD	SNR-Gd	CNR-Gd
5.54 ± 0.33 (4.1–6.0)	-0.13 ± 0.03 (-0.18 to -0.12)	0.18 ± 0.04 (0.14 to -0.23)	22.67 ± 1.66 (10.8 to 24.5)	22.00 ± 1.66 (21.5 to 25.2)

*SD* Standard deviation

Values are expressed as mean ± SD (min–max)

### Lumen area at the noncalcified level: conventional imaging compared to color Gd K-edge imaging

In the absence of calcifications, the mean lumen area was significantly different between conventional and color Gd K-edge images at 22.83 ± 0.12 mm^2^ and 21.03 ± 0.25 mm^2^, respectively (*p* < 0.001). For the conventional reconstruction, the overestimation of the physical reference lumen area (*i.e*., 19.63 mm^2^) was at 16%. For the color Gd K-edge reconstruction, this overestimation was reduced to 7%.

### Lumen area at the calcified plaque level

**Fig. 3:**
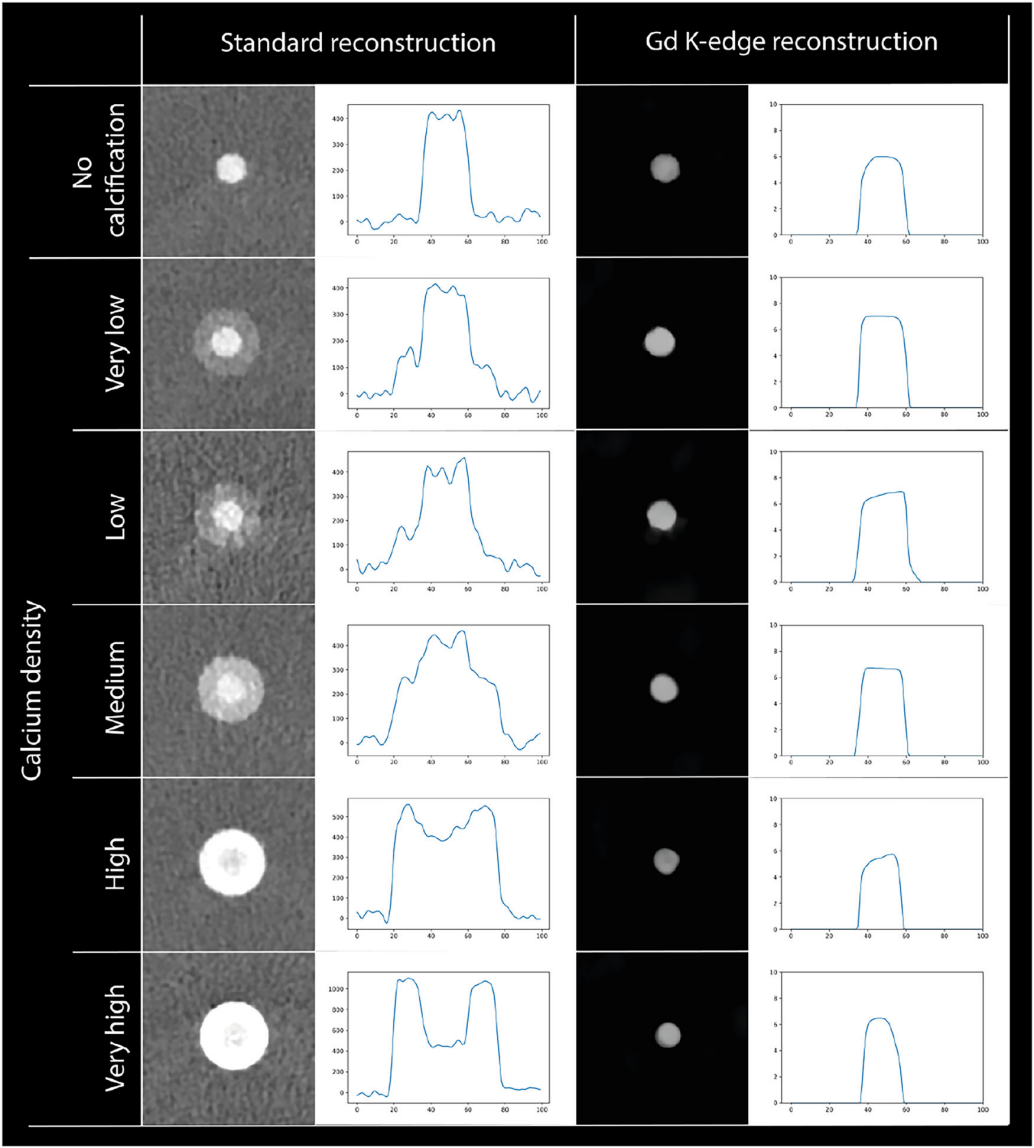
Standard conventional and color Gd K-edge images in combination with a Gd-based contrast agent for a noncalcified plaque and all five calcification densities. For both reconstructions, the image data (left) and a profile plot through the center of the lumen (right) are shown. For both profile plots, the x-axis shows the distance, while the y-axis shows the CT number (HU) or the Gd-concentration (mg/mL), for the conventional and K-edge reconstruction, respectively. For the CT image data, a window-width/window-level setting of 750/90 HU was used. For the color Gd K-edge image data, the values represent a minimum of 0 until a maximum of 10 mg/mL of Gd

**Fig. 4:**
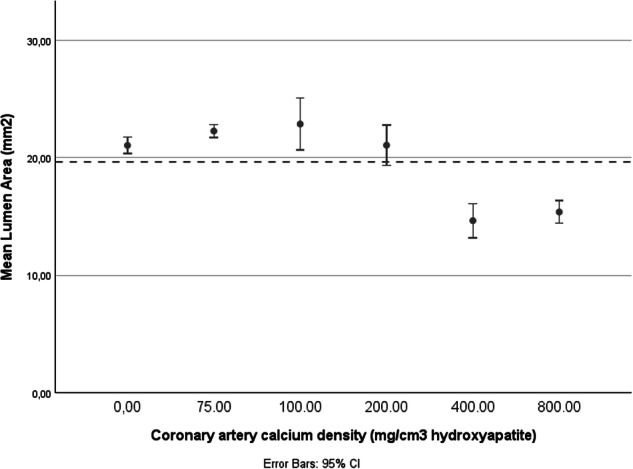
For each calcium density, designated as the non-plaque coronary artery artery (0 mg/cm^3^ HA), very low (75 mg/cm^3^ HA), low (100 mg/cm^3^ HA), medium (200 mg/cm^3^ HA), high (400 mg/cm^3^ HA), and very high (800 mg/cm^3^ HA) calcium density, the lumen area (mm^2^) was measured. The dotted line shows the reference lumen area. HA, Hydroaxyapatite

**Fig. 5:**
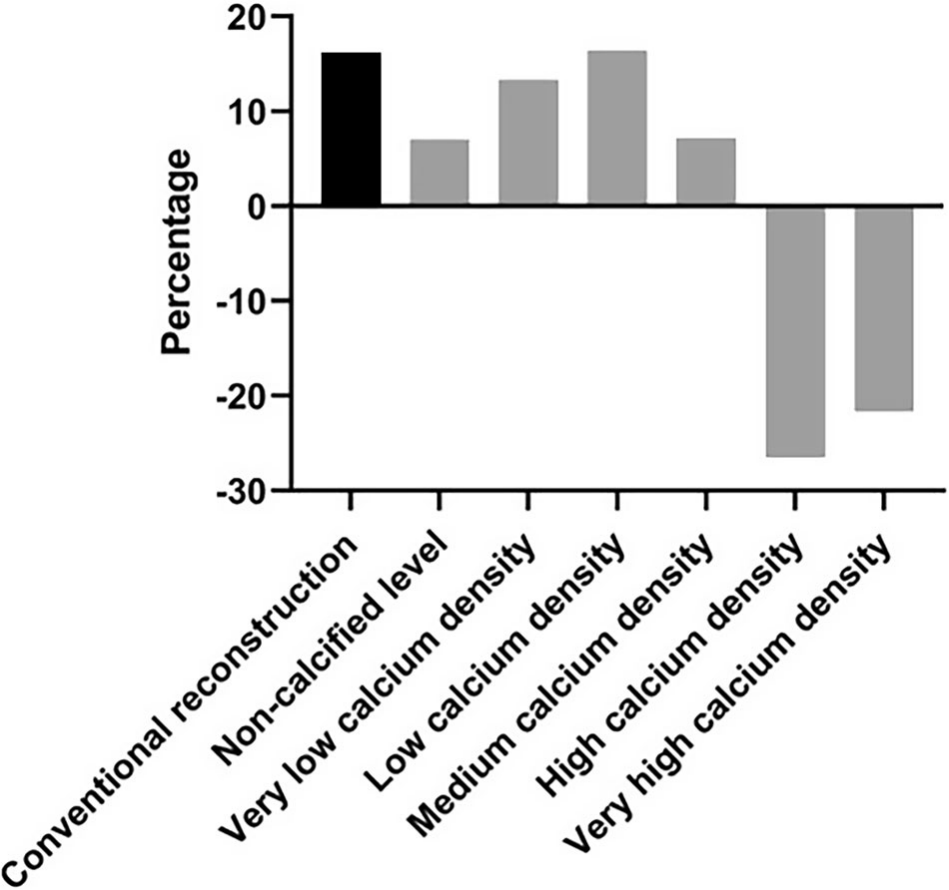
The percentage of over- or underestimation was calculated for conventional reconstruction (black bar) at the noncalcified level, and K-edge reconstructions at the noncalcified level and for each calcium density, designated as the very low (75 mg/cm^3^ HA), low (100 mg/cm^3^ HA), medium (200 mg/cm^3^ HA), high (400 mg/cm^3^ HA), and very high (800 mg/cm^3^ HA) calcium density (gray bars). HA, Hydroaxyapatite

For the conventional reconstructions, calcium and Gd CT numbers were comparable as expected, which did not allow for objective lumen assessment in the presence of calcifications (Fig. 3). Only color Gd K-edge images enable specific lumen assessment, as the calcium signal was not present anymore. This resulted in physical lumen assessment of 22.25 ± 0.43 mm^2^, 22.85 ± 1.77 mm^2^, 22.85 ± 1.77 mm^2^, 14.63 ± 1.17 mm^2^, and 15.37 ± 0.78 mm^2^ for very low, low, medium, high, and very high-density calcifications (Fig. 4). For the three lowest densities, the physical lumen was overestimated up to 16.4%, while an underestimation up to 26.5% of the physical lumen size was shown for the two highest density calcifications, as compared to the reference (Fig. 5). For the different calcification densities, the differences between lumen areas were significant (*p* < 0.001).

## Discussion

In the present study, color Gd K-edge imaging outperformed the evaluation of coronary artery lumen in the presence and in the absence of calcifications using the SPCCT system in comparison to conventional imaging, in combination with a humanly approved Gd-based contrast agent. First, color Gd K-edge imaging enabled Gd quantification in the presence and in the absence of calcifications. Second, color Gd K-edge imaging improved image quality, via means of its SNR and CNR. Third, color Gd K-edge imaging improved lumen area measurement in comparison to conventional images, even at the level of calcified plaque.

In the present study, color Gd K-edge imaging facilitates quantification of the exact concentration of the contrast agent via the means of specific detection and characterization of one specific element. In terms of accuracy, there is a very good correlation between the Gd concentration measured on color Gd K-edge imaging and the one injected, as previously shown with different systems [9, 16, 19, 27, 28]. But some discrepancies are noted, particularly in the presence of high-density calcifications. When the density of calcification increases, the linear attenuation curve of hydroxyapatite within the scanner’s energy range approaches that of Gd. Notably, for a density of 400 mg/cm³, the linear attenuation value of hydroxyapatite is really close to that of Gd at its K-edge energy (*i.e*., around 50 keV). This similarity may affect the accuracy of material decomposition, and might explain the observed lower Gd concentrations in the region with high-density calcifications compared to those with lower densities. These findings highlight a potential limitation of material decomposition, wherein overlapping material attenuation spectra can significantly compromise image quality and diagnostic accuracy. As demonstrated by Vecsey-Nagy et al, virtual non-iodine reconstructions in coronary CT angiography (CCTA) resulted in an 18.3% false-negative reclassification rate, indicating that lower scores were associated with reduced diagnostic performance [29]. This limitation may be attributed, in part, to spectral overlap, which impairs accurate material differentiation. Importantly, both Gd and iodine exhibit energy spectra that can interfere with precise image reconstruction and tissue characterization. In terms of iodine, the inability to identify the K-edge of iodine is not due to the detector’s capability, which can detect photons down to 30 keV, but is primarily attributed to the significant attenuation of photons at these low energies. Identifying the iodine K-edge requires observing an attenuation jump at 33.2 keV, specifically comparing the attenuation above this threshold (*e.g*., at 33.45 keV) to that in the 30–33 keV range. Data from the normalized tube spectrum after passing through 10 cm of water indicates that the signal below 33 keV is negligible. This attenuation makes it impossible to clearly identify the iodine K-edge after traversing human tissue. Furthermore, even in the absence of water, distinguishing the K-edge remains unfeasible due to the limited 3 keV energy bin, which does not provide sufficient photon statistics. These spectral considerations should be carefully evaluated in the development of future CT hardware and software updates.

In terms of SNR and CNR improvement, our findings align with previous results, as color Gd K-edge imaging produces target material-specific images, with inherent high SNR, as shown both in phantom, animal, and recent Human studies [9, 16, 19, 20]. In addition, Color Gd K-edge of Gd images are sharper, element-focused, and of relatively low image noise, thereby enhancing both CNR and SNR.

Importantly, our study was designed to target a specific HU threshold for Gd rather than aiming for exact concentration values. This decision was guided by clinical practice, where iodine-based contrast agents are typically required to achieve attenuation levels between 350 and 450 HU to meet diagnostic standards. While this approach does not allow for precise determination of Gd concentrations, it more accurately reflects real-world clinical scenarios. Furthermore, the algorithm employed for data analysis in this study operates based on CT numbers thresholds rather than contrast agent concentrations, which further justifies the chosen methodology. Kravchenko et al have presented findings on Gd-based coronary CTA, demonstrating that although Gd-enhanced CT scans can be acquired with photon-counting CT (PCD-CT) protocols, their feasibility is still limited. [30]. Notably, the PCD-CT system used in this study does not incorporate energy bins, which lay the ground for color Gd K-edge imaging. Instead, it relies solely on analysis across different monoenergetic levels, which is not specific to Gd, resulting in suboptimal analysis. However, as shown by Graf et al, patient scanning with Gd is feasible at the standard dose, when monoenergetic reconstruction is applied, underlining that Gd scans should be performed whenever iodinated contrast is contraindicated [22].

Recently, Holmes et al presented a study performed on a novel deep silicon-based spectral CT detector to investigate its performance in material differentiation [27]. As shown, the spectral CT scanner enabled differentiation between iodine and Gd, which was not possible when a conventional kV switching scanner was used. However, surprisingly, when looking at the CT number of Gd at different energy levels, it does not reflect the theoretical values for virtual monoenergetic levels. Similar to our results, Gd K-edge improved lumen analysis, as compared to conventional reconstructions. However, when looking at the images, the Gd map presented by Holmes’s group does not enable one to clearly differentiate between the Gd and calcification, which most probably might have hampered the results. In our study, in turn, when color Gd K-edge is applied, only Gd is visible, which enables clear differentiation between the two materials.

As color Gd K-edge imaging effectively isolates the target element, providing a clear visualization of Gd, it significantly improves the quantification of lumen area compared to conventional images. Boccalini et al demonstrated that vessel diameters calculated from color Gd K-edge images were comparable to those from conventional analyses. In the present study, lumen area analysis from color Gd K-edge images at the noncalcified level was superior to that from conventional images [19]. However, it is important to note that the arterial lumen diameter analyzed in the present study was larger. In addition, the lumen area was overestimated at the level of small and medium-density calcifications; nevertheless, a similar overestimation was observed with conventional reconstructions in the absence of calcifications. The underestimation of lumen area at the level of high-density calcifications was most probably due to persisting blooming artifacts. However, calcification-associated blooming artifacts are subjectively less pronounced when reconstructed using the color Gd K-edge imaging compared to conventional reconstruction methods [31].

The use of Gd-based contrast agents CT, although currently off-label, holds potential for enabling cardiac CT imaging in patients with contraindications to magnetic resonance scanning for myocardial tissue characterization. Another potential application includes the use of Gd in coronary CTA for patients who cannot receive iodinated contrast agents, for example, due to allergies. In addition, current developments in nanobiotechnology may offer new opportunities by utilizing optimized Gd chelates for vascular imaging, as demonstrated by Hernandez-Fajardo et al [14].

In this study, we showed that an ultra-small rigid platform can assess the arterial lumen similarly to gadoteric acid while increasing blood pool image enhancement by 20%. This innovation could extend beyond arterial imaging to enable more effective venous phase imaging, addressing a longstanding but previously unrealized goal in CT venography [19]. Moreover, knowing that the Gd K-edge energy is within the spectral limits of SPCCT, it may enable not only improved artery lumen analysis, but also lesion or tumor identification and further diagnosis. However, despite the promising theoretical advantages of Gd in CT, it is important to emphasize that, at present, this application remains hypothetical. Therefore, clinical studies implementing Gd as a CT contrast agent for SPCCT imaging are needed, to explore the potential of Gd in real clinical scenarios.

Our study has several limitations. Firstly, the SPCCT system used in our study is a prototype scanner. Secondly, the impact of cardiac motion was not evaluated in this static phantom study, which may not reflect the coronary CTA results. Nevertheless, the findings can be extrapolated not only to the analysis of coronary arteries but also to other arterial vessels [27]. The primary aim of our study was to explore the relationship between Gd and calcium within the arterial lumen, as well as the role of spectral imaging in characterizing this interaction. Importantly, these findings can be extended to different vascular territories, since atherosclerosis is a systemic process affecting all arteries throughout the body. This insight provides a potential pathway for translation into clinical practice. Nevertheless, further studies with a dynamic phantom will be needed to evaluate the impact of coronary artery motion on K-edge imaging practice. Interestingly, high-density calcification influences the calculation of Gd concentration, indicating the need for further improvement of the spectral forward model. Third, as we aimed for a certain attenuation of Gd (a concentration reaching 400 HU at 70 keV, which reflects the clinical range for CCTA when iodine is used as a contrast agent), not the concentration, the concentration of Gd is not known, and as a result, we could not verify the calculation from color Gd K-edge imaging. Fourth, as the K-edge of iodine is outside of the CT clinical range, the analysis of the K-edge of iodine was not available, and therefore, the comparison between iodine and Gd was not performed in this study. However, as the size of the phantom is known, the overall performance of the Gd K-edge can be analyzed. In addition, the size of calcifications was relatively large compared to calcifications usually visible *in vivo*. Moreover, the artery lumen was relatively large and mostly comparable to the left main artery.

High-resolution color Gd K-edge imaging dedicated to Gd enabled specific coronary CTA lumen imaging in both calcified and noncalcified arteries, in comparison with conventional imaging. With calcifications present, only color Gd K-edge imaging allowed for objective lumen assessment, while accuracy was dependent on the presence and density of calcium.

## Data Availability

The data that support the findings of this study are available from the corresponding author upon reasonable request.

## Abbreviations

CCTA: Coronary computed tomography angiography
CNR: Contrast-to-noise ratio
CT: Computed tomography
SD: Standard deviation
SNR: Signal-to-noise ratio
SPCCT: Spectral photon-counting computed tomography
